# Genetic and phenotypic spectrum of Chinese patients with epilepsy and photosensitivity

**DOI:** 10.3389/fneur.2022.907228

**Published:** 2022-08-02

**Authors:** Yue Niu, Pan Gong, Xianru Jiao, Zhao Xu, Yuehua Zhang, Zhixian Yang

**Affiliations:** Department of Pediatrics, Peking University First Hospital, Beijing, China

**Keywords:** photosensitive epilepsy, genes, electroencephalography, photoparoxysmal response, photoconvulsive response

## Abstract

**Objective:**

To determine the contribution of genetic etiologies in epilepsy with photosensitivity.

**Methods:**

A total of 35 epileptic patients with genetic photosensitivity from January 2019 to May 2021 were analyzed.

**Results:**

Pathogenic variants were identified in 35 patients, including *SCN1A*(7) *CHD2*(6), *TPP1*(3), *SYNGAP1*(3), *GABRA1*(2), *GABRG2*(1), *KCTD7*(1), *MFSD8*(1), *KCNC1*(1) *GBA*(1), *CACNA1A*(1), *KCNMA1*(1), *FLNA*(1), *SZT2*(1), *SLC2A1*(1), 5q33.2-34del(1), and mitochondrial variants(3). The predominant epileptic syndrome was progressive myoclonus epilepsy (PME) and Dravet syndrome, while the most common seizure type in both spontaneous seizures and photoconvulsive response (PCR) was myoclonic seizures. The abnormal EEG background and brain MRI were mainly seen in the PME patients. In PME, initial low-frequencies (1–6 Hz) photosensitivity was observed in 70% (7/10) of patients. Among the other patients, 12 patients (48.0%, 12/25) had photosensitivity at initial low -frequencies and 12 patients (48.0%, 12/25) had photosensitivity at initial middle frequencies (6–20 Hz). At the 1-year follow-up, 77.7% (21/27) still remained photosensitive.

**Conclusion:**

The most common genes for epilepsy with genetic photosensitivity are *SCN1A* and *CHD2*, and the most common syndromes are PME and Dravet syndrome. *MFSD8, KCNMA1, SZT2, FLNA*, and *SLC2A1* variants might be candidate genes for photosensitivity. PPRs at initial low-frequencies may be a marker of PME, and the most typical feature of genetic photosensitivity may be low- or middle- frequencies induced PPRs. Photosensitivity in epilepsy with genetic photosensitivity may be difficult to disappear in a short period of time.

## Graphical Abstract

EEG, electroencephalography; IPS, Intermittent Photic Stimulation; PPR, photoparoxysmal response; PCR, photoconvulsive response. 
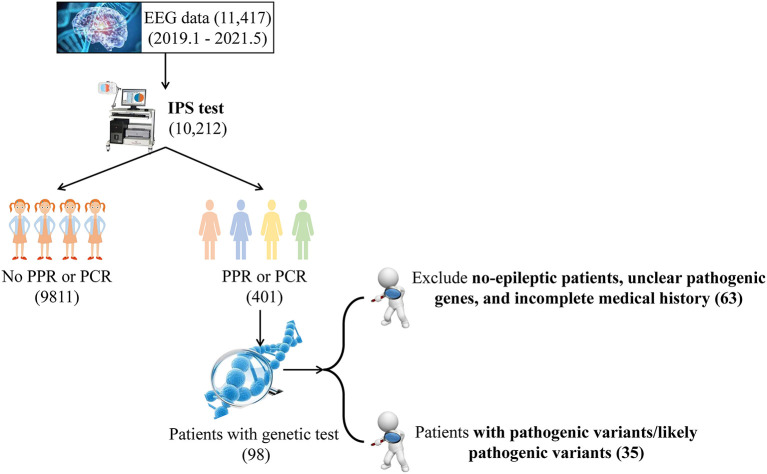

## Introduction

Photosensitivity usually manifests as abnormal and characterized electroencephalography (EEG) with a paroxysmal reaction to intermittent photic stimulation (IPS), which is a heritable abnormal cortical response to light stimuli (1). In previous studies, the prevalence of photosensitivity in non-epileptic subjects ranged from 0.5 to 8.9% of the population and 10–20% of children with epilepsy (2). With technological advancement, human exposure to photographic stimuli through new media such as television and video games has increased, making photosensitivity more prevalent.

Epilepsy with photosensitivity comprises of a heterogeneous group of epileptic conditions including genetic generalized epilepsy (GGE) syndromes, Dravet syndrome, Lennox-Gastaut syndrome, and epilepsy with myoclonic absences (3). Photosensitivity is also apparent in progressive neurodegenerative disorders such as neuronal ceroid lipofuscinoses (NCL), Lafora's disease, Unverricht Lundborg disease and mitochondrial disorders (such as myoclonus epilepsy and ragged red fibers) (4).

Epilepsy with photosensitivity has a complex genetic architecture with several linked loci, such as chromosomes 6, 7, 13, and 16 (5). In 2015, a study confirmed the *CHD2* variant as a risk factor for photosensitivity through a functional study of chd2 knockout in zebrafish larvae (6). In 2016, a cohort of patients with *GABRA1* mutations was analyzed and 36% of them had a generalized photoparoxysmal response (PPR) within a mixture of epileptic disorders, suggesting that the pathophysiology of PPR might be shared with other disorders of GABA inhibition (7). Photosensitivity is considered a characteristic of Dravet syndrome, and many cases of *SCN1A* mutations have been described with photosensitivity (8). Although there is a large genetic component to epilepsy with photosensitivity, many genes have not been identified. Therefore, we sought to explore the contribution of genetic etiologies in epilepsy with photosensitivity in our center.

## Methods

### Ethics approval and consent to participate

This study was approved by the Biomedical Research Ethical Committee of Peking University First Hospital. The individuals' parents in this manuscript have given written informed consent to publish the case details.

### Participants

We screened all patients from the Department of Pediatrics, Peking University First Hospital from January 2019 to May 2021, and the specific screening flow chart is shown in Graphical abstract. First, photosensitivity was queried in the EEG database of the Department of Pediatrics, Peking University First Hospital for the past few years. Second, patients with photosensitivity identified in the first step were screened by the following clinical criterias: (a) seizure, (b) PPR or photoconvulsive response (PCR), and (c) having taken a genetic assessment, including trio-based targeted gene panels testing (Supplementary Table 1) or whole-exome sequencing, copy-number variant (CNV), and mitochondrial genome sequencing. The exclusion criteria were as follows: (a) no- seizure patients, and (b) unclear pathogenic variants (c) incomplete medical history. Excluding five patients with incomplete data (two with Dravet syndrome carrying *SCN1A* variants, one with *SCN8A* variants, one with methylmalonic acidemia and one with carnitine deficiency), a total of 35 patients were eventually enrolled in the study.

IPS was performed in a dimly lit environment using a round lamp with a diameter of 10 cm. IPS was followed by an increase in the stimulation frequency from 1 Hz to 20 Hz, and then a decrease from 60 to 20 Hz, which was an increase of 2 Hz or a reduction of 10 Hz, respectively. Five-second trains of flashes for each frequency were delivered, at intervals of 10 s. When IPS induced seizures, the IPS test should be stopped immediately. Photosensitivity was defined as PPR and/or PCR in response to IPS. In our study, few samples had type I (spikes within the occipital rhythm), so we classified type 1 and type 2 (parieto-occipital spikes with a biphasic slow wave) of the classification criteria as grade I (posterior EDs). The epileptiform discharges (EDs) of PPR/ PCR were graded into three grades: posterior EDs (grade I), starting temporoparietooccipital and spreading to frontal regions (grade II), and generalized EDs (grade III) (9).

### Pathogenicity analysis and statistics analysis

Genomic DNA was extracted from peripheral blood lymphocytes using standard protocols. Variants segregated within a family in an autosomal recessive or X-linked manner were ruled out if the max allele frequency (ExAC or gnomAD) was >0.001. *De novo* variants were ruled out if the max allele frequency (ExAC or gnomAD) was > 0.0001. For candidate genes, variants were predicted by Mutation Taster,^1^ Polyphen-2,^2^ SIFT,^3^ Splice site prediction,^4^ CADD,^5^ and Provean (see text footnote 3). Variants pathogenicity were interpreted according to the American College of Medical Genetics (ACMG) guidelines and further confirmed by Sanger sequencing (10). Kaplan-Meier plots and log-rank analysis were used to calculate the age of photosensitive epilepsy patients at the time of first seizure, first EEG, first IPS and first photosensitivity. The χ^2^-test was used to compare the relationships among the PME, Dravet syndrome and unclassified epileptic encephalopathy. A *p*-value < 0.05 was considered statistically significant. SPSS software V.25.0 was used for data analysis.

## Results

Our study enrolled 35 patients, including 10 PME patients with seven monogenic and three mitochondrial DNA (mt-DNA) variants, seven patients with *SCN1A* variants, six patients with *CHD2* variants, 11 patients with other monogenic variants, and one patient with a CNV. The detailed clinical characteristics of the 35 patients were summarized in Table 1.

**Table 1:** Clinical characteristics of 35 pathogenic/likely pathogenic epileptic patients with photosensitivity.

**No**.	**Variant information**	**Epilepsy syndrome**	**Seizure types**	**EEG background**	**Interictal EDs**	**Eye states of PPR (Hz)**	**Grade PPR**	**Eye states of PCR (Hz)**	**Grade PCR**	**Brain MRI**	**Associated neurological features**
Patient 1	TPP1 c.[1424C>T, c.1222-1224del] (p.Ser408del; Ser475Leu;)	PME	MS	Abnormal (slow waves)	Generalized/multifocal (dominated by the posterior)	EO (1–30)	II	EO (14–40)	III	Abnormal signal in the right frontotemporal lobe	CNL2, GDD/ID
Patient 2	TPP1 c.515delG (p.Gly172Aspfs*11)	PME	MS, FS	Abnormal (slow waves)	Generalized/multifocal (dominated by the anterior and posterior)	-	-	EO (1–50)	III	White matter dysplasia and progressive parenchymal atrophy	CNL2, GDD/ID, hypotonia, tremor and ataxia
Patient 3	TPP1 c.177_180delAAGA (p.Glu59Aspfs*21)	PME	MS, FS	Normal	Focal (dominated by the occipital area)	EO (6–14)	I	EO (16)	I	Progressive parenchymal atrophy	CNL2, GDD/ID and ataxia
Patient 4	KCTD7 c.[458G>A; 533C>T] (p.Arg153His; Ala178Val)	PME	MS, FS, AtS, aAS, SE (FS, aAS)	Abnormal (slow waves)	Multifocal	EO (1–60 Hz)	I	-	-	Normal	CNL14, GDD/ID, esotropia, hypertonia and right hemiplegia
Patient 5	MFSD8 c. [1351-G>A, 557T>G] (p.Phe186Cys)	PME	MS, FS	Abnormal (slow waves)	Generalized/multifocal (dominated by the posterior)	EO (4–30)	I	-	-	Right anterior cingulate gyrus cortical dysplasia	CNL7, GDD/ID and ataxia
Patient 6	KCNC1 c.595G>A (p.Arg320His)	PME	MS, GTCS, aAS	Normal	Generalized/multifocal	ECL (10–20)	III	-	-	Normal	GDD/ID, unstable walking
Patient 7	GBA c.[680A>G c.1448T>C] (p.Asn227Ser, p.Leu483Pro)	PME	MS, FS, GTCS SE (MS)	Abnormal (diffuse fast waves)	Rolandic	-	-	EO (6–60) EC (1–25) ECL (2–25)	III	White matter dysplasia progressive parenchymal atrophy	Gaucher disease, GDD/ID, wave-like changes in both eyes, refractive errors, hypotonia and ataxia
Patient 8	Mt DNA m.10158T>G	PME	MS, EM, aAS, SE (FS)	Abnormal (slow waves)	Generalized/multifocal (dominated by the posterior)	EO (12–30) ECL (10–60)	I, III	EO (20–25) ECL (10–25) ECL (18–30)	I, III	Abnormal signals in bilateral basal ganglia, thalamus, and brainstem	Leigh-MELAS, GDD/ID, strabismus, decreased vision, restricted right eye abduction tremor, decreased muscle volume
Patient 9	Mt DNA m.3243A>G	PME	MS FS SE (FS)	Abnormal (slow waves)	Rolandic (ESES60%)	EO (16–18)	I	EO (12–30)	I	Normal	Mitochondrial encephalopathy, GDD/ID, hypotonia, short stature
Patient 10	Mt DNA m.3243A>G	PME	MS, FS	Abnormal (slow waves)	Multifocal	EO (20–30) EC (1–60) ECL (1–30)	III	-	-	Abnormal signal in bilateral parieto-occipital temporal cortex and subcortex, progressive parenchymal atrophy	MELAS, GDD/ID, visual field defect, short stature
Patient 11	CHD2 c.2644G>T (p.Val882Phe)	LGS	FS, MS, TS (generalized)	Normal	Generalized	EO (25)	III	-	-	Normal	GDD/ID
Patient 12	CHD2 c.4051_4052del (p.Lys1351Serfs*11)	LGS	GTCS, FS, aAS, AtS	Normal	Generalized/multifocal (dominated by the posterior)	EO (4–50) EC (1–50) ECL (1–20)	I	-	-	Normal	GDD/ID
Patient 13	CHD2 c.4278delG (p.Lys1426Asnfs*51)	NEE	MS	Normal	Generalized/multifocal (dominated by the anterior)	-	-	EO (10–14)	III	Normal	GDD/ID, autistic behavior
Patient 14	CHD2 c.1719G>A (p.T573i)	NEE	MS, aAs, AtS, TS (generalized) CS (generalized)	Normal	Generalized/multifocal	EO (10–14)	I	-	-	Normal	Sever GDD/ID
Patient 15	CHD2 c.3454C>T (p.Arg1152Trp)	JS	EM, MS	Normal	Generalized	EO (8–50)	I, III	-	-	Normal	-
Patient 16	CHD2 c.4156_4157insA (p.Ser1386Lysfs*23)	JS	GTCS, EM	Normal	Generalized	EC (20–25) ECL (18–20)	III	-	-	Normal	GDD/ID
Patient 17	SYNGAP1 c.3061C>T (p.Gln1021*)	NEE	Ats	Normal	Rolandic	EO (14–60)	I	-	-	White matter dysplasia	GDD/ID
Patient 18	SYNGAP1 c.1984C>T (p.Gln662*)	JS	EM, aAS	Normal	Generalized/multifocal	EO (1–60)	I, III	-	-	Normal	GDD
Patient 19	SYNGAP1 c.1514delA (p.Tyr505Serfs*22)	NEE	aAs, FS	Normal	Generalized/occipital	-	-	EO (14)	III	Normal	ID
Patient 20	GABRA1 c.644T>C (p.Leu215Pro)	Dravet	Febrile seizure, FS, MS, GTCS	Normal	Generalized/multifocal (dominated by the posterior)	-	-	EO (1–60)	I, III	Normal	GDD/ID
Patient 21	GABRA1 c.228T>G (p.Ser76Arg)	NEE	Febrile seizure, FS	Normal	Multifocal	EO (8–25)	I	-	-	Normal	GDD/ID
Patient 22	GABRG2 c.242T>C (p.Leu81pro)	CAE	Febrile seizure, aAS	Normal	Generalized	EO (6–50)	III	-	-	Normal	-
Patient 23	CACNA1A c.2039-2040del (p.Gln680ArgfsTer100)	JAE	AS	Normal	Generalized/occipital	-	-	EC (10) ECL (10)	III	Normal	GDD/ID
Patient 24	KCNMA1 c.2984A>G (p.Asn995Ser)	GGE	MS	Normal	Generalized/multifocal	EO (8–30)	IIII	-	-		Paroxysmal dyskinesia, GDD/ID
Patient 25	SLC2A1 c.1199G>A (p.Arg400His)	GLUT1DS	MS, aAS	Normal	Generalized	EO (1–60)	III	EO (2–30)	III	Normal	GLUT1-DS, GDD/ID
Patient 26	FLNA c.1243G>A (p.Glu415Lys)	NEE	MS, FS, aAS	Abnormal (slow waves)	Multifocal	EO (1–30)	I	-	-	Normal	Intestinal atresia, GDD/ID, ataxia
Patient 27	SZT2 c.[5705T>C, 2887A>G] (p.Val1902Ala, p.Lys953Glu)	NEE	aAS, spasm, FS	Abnormal (slow waves)	Generalized/multifocal (dominated by the posterior)	EO (1–60)	I, III	EO (25–30)	II	White matter dysplasia	GDD/ID
Patient 28	SCN1A c.4554dupA (p.Pro1519Thrfs*18)	Dravet	Febrile seizure, FS, aAs	Normal	Multifocal (dominated by the posterior)	EO (1–60)	I	-	-	Normal	GDD
Patient 29	SCN1A c.1197_1198delCA (p.Met400Aspfs*49)	Dravet	Febrile seizure FS, GTCS SE (FS)	Normal	Generalized/ multifocal (dominated by the anterior)	EO (12) EC (10–30) ECL (60)	III	-	-	Normal	GDD/ID, autistic behavior
Patient 30	SCN1A c.3836_3837delAT (p.Tyr1279Phefs*14)	Dravet	Febrile seizure MS, FS GTCS/FGTCS SE (FS)	Normal	Generalized/multifocal (dominated by the posterior)	EO (6–60)	I	-	-	Normal	GDD/ID
Patient 31	SCN1A c.2831T>C (p.Val944Ala)	Dravet	Febrile seizure FS, GTCS MS, SE (FS)	Abnormal (slow waves)	Generalized	EO (1–14)	III	EO (14)	III	Reduced white matter volume in the brain	Sever GDD
Patient 32	SCN1A c.1088C>T (p.Thr363Ile)	Dravet	Febrile seizure FS, MS, SE(FS)	Normal	Generalized/multifocal	EO (1–60)	III	EO (1–60)	III	Normal	GDD
Patient 33	SCN1A c.902delA (p.Asn301Metfs*5)	Dravet	Febrile seizure FS, MS, SE(FS)	Normal	Multifocal (dominated by the posterior)	EO (2–30)	III	-	-	Normal	GDD/ID
Patient 34	SCN1A c.4762T>G (p.Cys1588Gly)	Dravet	Febrile seizure MS, FS, FGTCS SE (FS)	Normal	Generalized/ multifocal	EO (18–20)	III	EO (25)	III	Normal	GDD/ID
Patient 35	5q33.2-34del	NEE	Febrile seizure FS	Normal	Generalized (dominated by the posterior)	ECL (12–20)	III	-	-	Normal	Sever GDD

NA, not available; PME, progressive myoclonus epilepsy; LGS, Lennox-Gastaut syndrome; NEE, unclassified epileptic encephalopathy; JS, jeavons syndrome; CAE, childhood absence epilepsy; JAE, juvenile absence epilepsy; GGE, genetic generalized epilepsy; GLUT1DS, glucose transporter type 1 deficiency syndrome; MS, myoclonic seizure; FS, focal seizure; AtS, atonic seizure; aAS, atypical absence seizure; GTCS, generalized tonic-clonic seizure; EM, eyelid myoclonic; CS, clonic seizure; TS, tonic seizure; SE, status epilepticus; FGTCS, focal secondary generalized tonic-clonic seizures; EEG, electroencephalogram; EDs, epileptiform discharges; PPR, photoparoxysmal response, PCR, photoconvulsive response; EO, eye opened; EC, eye closed; ECL, eye closure; I, temporoparietooccipital discharges; II, starting temporoparietooccipital and spreading to frontal regions; III, generalized discharges; MRI, magnetic resonance; GDD, global developmental delay, ID, intellectual disability; CLN, ceroid lipofuscinosis type.

### Clinical characteristics

**Figure 1:**
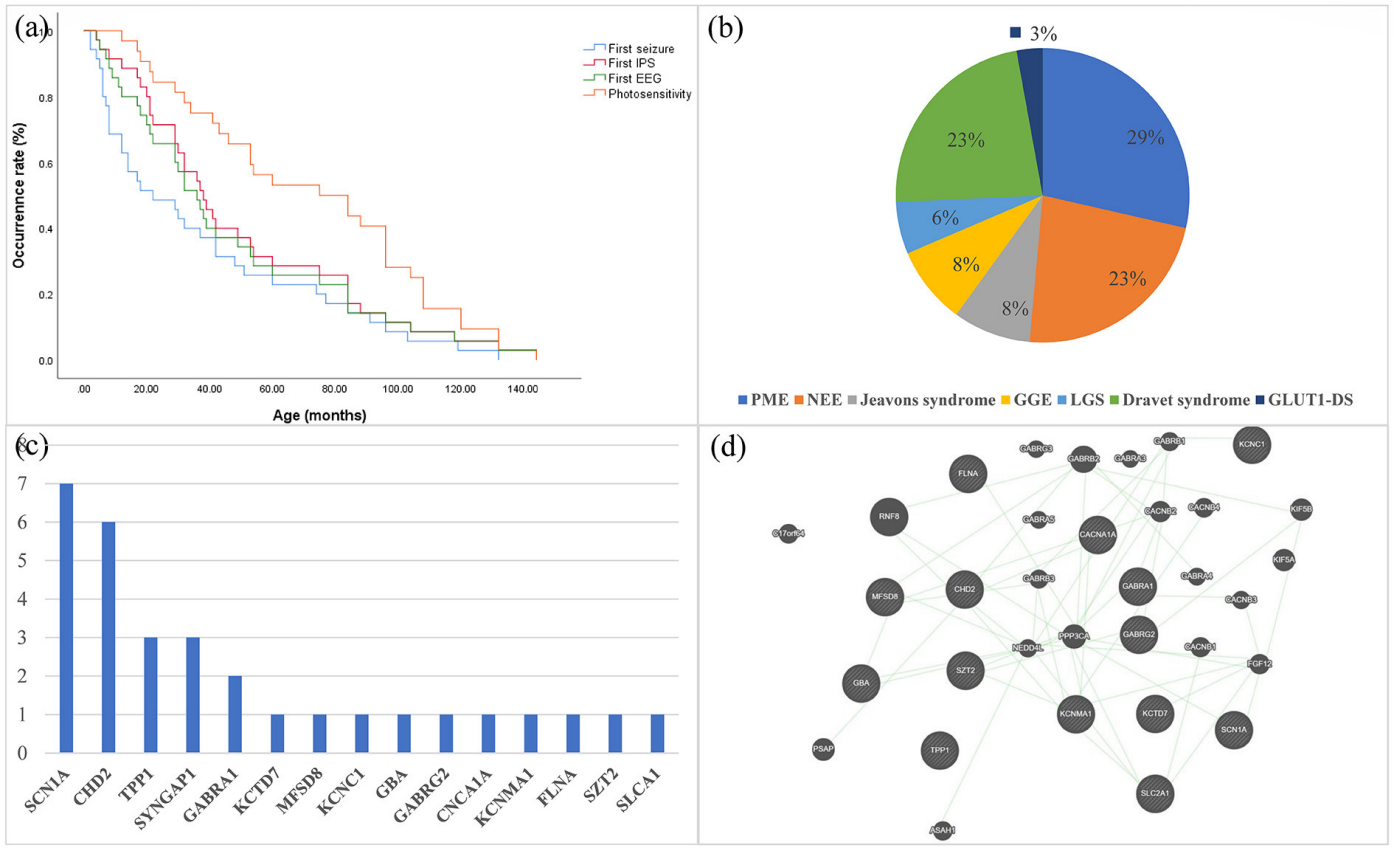
**(a)** Kaplan–Meier estimates of the age of all patients at the time of first seizure, first EEG, first IPS, and first PPR/PCR are shown. **(b)** Distribution of syndromes in epileptic patients with genetic photosensitivity. **(c)** Frequency distribution of monogenic variants that are pathogenic or likely pathogenic for photosensitivity. **(d)** Prediction of the genetic interactions performed by Genemania (accessed on 11 March 2021). EEG, electroencephalography; IPS, Intermittent Photic Stimulation; PPR, photoparoxysmal response; PCR, photoconvulsive response; PME, progressive myoclonus epilepsy; NEE, unclassified epileptic encephalopathy; LGS, Lennox-Gastaut syndrome; GGE, genetic generalized epilepsy.

There were 19 females in all patients. The mean age at first seizure for all patients was 37.8 months. The median age at first EEGs was 46.7 months, and the mean age at EEGs with first IPS was 60.7 months. The mean age at first photosensitivity was 69.1 months and 48.6% (17/35) presented photosensitivity at the first IPS (Figure 1a). A family history of epilepsy was reported in three of 35 probands, all of whom were second- or higher-degree family members. In addition, one patient's grandmother had motor neuron disease.

For the classification of epilepsy syndrome, 10 patients harboring the same or different variants were diagnosed with PME (*TPP1 n* = 3, *mt-DNA n* = 3, *KCTD7 n* = 1, *MFSD8, n* = 1, *KCNC1 n* = 1, and *GBA n* = 1), eight patients with Dravet syndrome (*SCN1A n* = 7, *GABRA1 n* = 1), eight patients with unclassified epileptic encephalopathy (*CHD2 n* = 2*, SYNGAP1 n* = 2, *GABRA1 n* = 1, *FLNA n* = 1, *SZT2 n* = 1, 5q33.2-34del *n* = 1), three with Jeavons syndrome (*CHD2 n* = 2, *SYNGAP1 n* = 1), three with GGE (*GABRG2 n* = 1, *CACN1A n* = 1, *KCNMA1 n* = 1), two with Lennox-Gastaut syndrome (*CHD2 n* = 2*)*, and one glucose transporter type 1 deficiency syndrome (GLUT1DS; *SLC2A1 n* = 1; Figure 1b). Among the three patients diagnosed with GGE, one had juvenile absence epilepsy, one had childhood absence epilepsy, and one had unclassified GGE.

### Spontaneous seizures

In all patients, the spontaneous seizure types were myoclonic seizures in 23 patients, focal seizures in 22, atypical absence seizures in 12, generalized tonic-clonic seizures in eight, eyelid myoclonic seizures in four, atonic seizures in four, spasm seizures in two, focal secondary generalized tonic-clonic seizures in two, generalized tonic seizures in one, and generalized clonic seizures in one patient. Notably, a total of 10 patients (six with Dravet syndrome and four with PME) presented with status epilepticus. In PME, four PME patients developed status epilepticus, where P4 presented two types of status epilepticus with focal seizures and atypical absence seizure, P7 presented myoclonic status epilepticus, and two patients (P8 and P9) had focal seizure status epilepticus with impaired consciousness. In Dravet syndrome, all six patients experienced focal seizure status epilepticus with impaired consciousness (Table 1).

### Brain magnetic resonance imaging (MRI)

Abnormal MRI was present in 28.6% (10/35) of patients. White matter dysplasia was identified in four patients (*TPP1 n* = 1, *GBA n* = 1, *SYNGAP1 n* = 1, *SZT2 n* = 1), with the *GBA* patient occurring after 2 years of the course, the *TPP1* patient after 5 months of the course, and the remaining patients at the first MRI. Progressive parenchymal atrophy appeared within 1–2 years of the disease course (*TPP1 n* = 2, *GBA n* = 1, mt-DNA *n* = 1). The remaining abnormal MRI findings included abnormal cortical signals (mt-DNA *n* = 2), reduced white matter volume (*SCN1A n* = 1), right anterior cingulate gyrus cortical dysplasia (*MFSD8 n* = 1), and abnormal signals in the right frontotemporal lobe (*TPP1 n* = 1), all of which were found on the first MRI.

### EEG

#### Background

An abnormal EEG background was identified in 11 patients, mainly seen in PME patients. The proportion of abnormal EEG backgrounds in patients with PME were significantly higher than those with Dravet syndrome and unclassified epileptic encephalopathy (*P* <0.001). In PME, eight patients had abnormal EEG backgrounds (seven with predominantly slow waves). The other three abnormal EEG backgrounds were all dominated by slow waves, in patients with *SCN1A, FLNA*, and *SZT2* variants.

#### Interictal EDs

Interictal EDs were observed in all patients. In PME, all patients had multifocal/focal EDs, while five also had generalized EDs. The distribution of multifocal/focal EDs showed that three patients had predominantly posterior EDs, three showed EDs in the Rolandic area, one had both posterior and anterior EDs, one had predominantly occipital EDs and two had no apparent dominant EDs. In *SCN1A*, only two patients had only multifocal EDs, while the remaining five patients had both generalized and multifocal EDs. In *CHD2*, all patients had generalized EDs, and three patients had simultaneous multifocal EDs. In the other genes, generalized and multifocal EDs were present in six patients, only generalized EDs were present in three patients, and only focal EDs were present in three patients.

#### IPS results

PPR was induced in 74.3% (26/35) patients. Most patients (65.4%, 17/26) had PPRs grade III; 3.8% (1/26) had a PPRs grade II; and 57.6% (15/26) had PPRs grade II.

PCR was induced in 42.9% (15/35) of patients. Seizure types in the PCR included myoclonic seizures in eight patients, focal seizures in three, atypical or typical absence seizures in two, eyelid myoclonic seizures in one, and focal secondary generalized seizures in one. PCR showed grade III in 80.0% (12/15) patients, grade I in 26.7% (4/15), and grade II in 6.7% (1/15) of patients.

**Figure 2:**
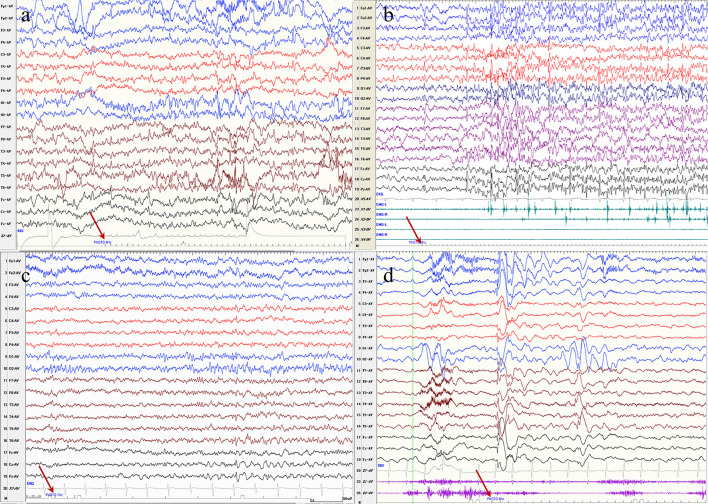
**(a)** Electroencephalography of a patient with progressive myoclonus epilepsy with a *KCTD7* variant demonstrates bioccipital spike-waves discharges in response to photic stimlation at 6 Hz. **(b)** Electroencephalography of a patient with progressive myoclonus epilepsy with a *GBA* variant demonstrates generalized spike-waves with a myoclouns seziure in response to photic stimlation at 8 Hz. **(c)** Electroencephalography of a patient with a Lennox-Gastaut syndrome with a *CHD2* variant demonstrates a bioccipital spike waves discharges in response to photic stimlation at 1 Hz. **(d)** Electroencephalography of a patient with Jeavons syndrome with a *SYNGAP1* variant demonstrates generalized spike waves or posterior discharges in response to photic stimlation at 4 Hz.

In PME, seven (70%, 7/10) patients had an initial response at low (1–6 Hz) IPS frequencies, and three patients (30%, 3/10) had an initial response at middle (6–20 Hz) IPS frequencies (Figures 2a,b) In other patients, 12 patients (48.0%, 12/25) had an initial response at low (1–6 Hz) IPS frequencies, and 12 patients (48.0%, 12/25) had an initial response at middle (6–20 Hz) IPS frequencies (Figures 2c,d). PME patients had a higher rate of low IPS frequencies than other patients (*P* > 0.05).

### Gene variants

We identified 36 variants in 15 genes, two mitochondrial variants and one CNV (Table 2). Of the 15 genes, nine are autosomal dominant (*KCNC1, CHD2, SYNGAP1, GABRA1, GABRG2*, CACNA1A, *SLC2A1, KCNMA1, SCN1A*), five are autosomal recessive (*TPP1, KCTD7, MFSD8, GBA, SZT2*), and one is X-linked (*FLNA*). Among the mt-DNA variants, the variant (m.3243A>G) was identified in two patients. P35 carries a novel deletion 5q33.2-34 with a mutation size of 9.53 Mb, which includes the genes *GABRA1, GABRB2*, and *GABRG2* related to photosensitivity.

**Table 2:** Pathogenicity analysis of monogenic variants in epilepsy with photosensitivity.

**Patient**	**Gene**	**Variant**	**Genome build (GRCh37)**	**Nucleotide change**	**Inheritance**	**EXAC allele frequency (v1.0)**	**Allele frequency in updated gnom AD (v2.1.1 and v3.1.1)**	**Reported in the Clinvar**	**ACMG classification**
1	TPP1 (NM_000391.4)	p.Ser475Leu	Chr11:6636403G>A	c.1424C>T	Heterozygous	<0.01%	<0.01%	Yes	P
1	TPP1	p.Ser408del	Chr11:6636715_6636717delACT	c.1222_1224delAGT	Heterozygous	0	<0.01%	No	LP
2	TPP1	p.Gly172Aspfs*11	Chr11:6638378_6638378delC	c.515delG	Homozygous	0	0	No	P
3	TPP1	p.Glu59Aspfs*21	Chr11:6640056_6640059delTCTT	c.177_180delAAGA	Homozygous	0	0	No	P
4	KCTD7 (NM_153033.5)	p.Arg153His	Chr7:66103383G>A	c.458G>A	Heterozygous	<0.01%	<0.01%	Yes	P
4	KCTD7	p.Ala178Val	Chr7:66103882C>T	c.533C>T	Heterozygous	<0.01%	<0.01%	Yes	P
5	MFSD8 (NM_152778)	p.Phe186Cys	Chr4:128861149A>C	c.557T>G	Heterozygous	0	0	No	LP
5	MFSD8	-	Chr4:128841992C>T	c.1351-1G>A	Heterozygous	0	<0.01%	No	P
6	KCNC1 (NM_001112741.2)	p.Arg320His	Chr11:17793600G>A	c.959G>A	*De novo*	0	0	Yes	P
7	GBA (NM_000157.4)	p.Asn227Ser	Chr1:155208006T>C	c.680A>G	Heterozygous	<0.01%	<0.01%	Yes	P
7	GBA	p.Leu483Pro	Chr1:155205043A>G	c.1448T>C	Heterozygous	<0.01%	<0.5%	Yes	P
11	CHD2 (NM_001271.4)	p.Val882Phe	Chr15: 93521530G>T	c.2644G>T	*De novo*	0	0	No	LP
12	CHD2	p.Lys1351Serfs*11	Chr15:93543785_93543786delAA	c.4051_4052delAA	*De novo*	0	0	No	P
13	CHD2	p.Lys1426Asnfs*51	Chr15:93545547delG	c.4278delG	*De novo*	0	0	No	P
14	CHD2	p.T573i	Chr15:93496803G>A	c.1719G>A	*De novo*	0	0	Yes	P
15	CHD2	p.Arg1152Trp	Chr15:93534746C>T	c.3454C>T	*De novo*	0	0	Yes	P
16	CHD2	p.Ser1386Lysfs*23	Chr15:93545425_93545426insA	c.4156_4157insA	*De novo*	0	0	No	P
17	SYNGAP1 (NM_006772.3)	p.Gln1021*	Chr6:33411390C>T	c.3061C>T	*De novo*	0	0	No	P
18	SNGAP1	p.Gln662*	Chr6:33409020C>T	c.1984C>T	*De novo*	0	0	No	P
19	SYNGAP1	p.Tyr505Serfs*22	Chr6:33406323_33406323delA	c.1514delA	*De novo*	0	0	No	P
20	GABRA1 (NM_001127644.2)	p.Leu215Pro	Chr5:161309648T>C	c.644T>C	*De novo*	0	0	No	LP
21	GABRA1	p.Ser76Arg	Chr5:161292676T>G	c.228T>G	*De novo*	0	0	No	LP
22	GABRG2 (NM_198904.4)	p.Leu81pro	Chr5:161520968T>C	c.242T>C	*De novo*	0	0	No	LP
23	CACNA1A (NM_00112722.2.)	p.Gln680Argfs*10000	Chr19:13414645_13414646delCT	c.2039_2040delAG	*De novo*	0	0	Yes	P
24	KCNMA1 (NM_002247.3)	p.Asn995Ser	Chr10:78651467T>C	c.2984A>G	*De novo*	0	0	No	P
25	SLC2A1 (NM_006516.4)	p.Arg400His	Chr1:43393355C>T	c.1199G>A	*De novo*	0	0	Yes	P
26	FLNA (NM_001110556.2)	p.Glu415Lys	Chr23:153594578C>T	c.1243G>A	His mother	<0.01%	<0.01%	No	LP
27	SZT2 (NM_015284)	p.Val1902Ala	Chr1:43900128T>C	c.5705T>C	Heterozygous	0	<0.01%	No	LP
27	SZT2	p.Lys963Glu	Chr1:43891578A>G	c.2887A>G	Heterozygous	<0.01%	<0.01%	Yes	P
28	SCN1A (NM_001165963.4)	p.Pro1519Thrfs*18	Chr2:166852549_166852550insT	c.4554dupA	*De novo*	0	0	No	P
29	SCN1A	p.Met400Aspfs*49	Chr2:166903459_166903460delTG	c.1197_1198delCA	*De novo*	0	0	No	P
30	SCN1A	p.Tyr1279Phefs*14	Chr2:166868661_166868662delAT	c.3836_3837delAT	*De novo*	0	0	No	P
31	SCN1A	p.Val944Ala	Chr2:166894401A>G	c.2831T>C	*De novo*	0	0	Yes	P
32	SCN1A	p.Thr363Ile	Chr2:166904219G>A	c.1088C>T	*De novo*	0	0	No	LP
33	SCN1A	p.Asn301Metfs*5	Chr2:166908291_166908291delT	c.902delA	*De novo*	0	0	No	P
34	SCN1A	p.Cys1588Gly	Chr2:166850746A>C	c.4762T>G	*De novo*	0	0	No	LP

Thirty-six monogenic variants were identified in 31 patients, including *SCN1A(7), CHD2(6), TPP1(3), SYNGAP1*(3), *GABRA1*(2), *GABRG2*(1), *KCTD7*(1), *MFSD8*(1), *KCNC1*(1) *GBA*(1), *CACNA1A*(1), *KCNMA1*(1), *FLNA*(1), *SZT2*(1), and *SLCA1*(1) (Figure 1c). The interactions of several genes were predicted by Genemania (Figure 1d). Among these monogenic variants, there were 20 missense variants, 11 frame-shifts variants, two nonsense variants, one splice site variant, one in-frame deletion variant, and one synonymous variant. The underlying channelopathy pathway accounted for 46.7% (7/15) of the monogenic variants, including ion channel genes (*SCN1A, KCTD7, KCNC1, CACNA1A, KCNMA1*) and ligand-gated ion channel genes (*GABRA1, GABRG2*). Notably, *KCNMA1, MFSD8, SZT2*, and *FLNA* variants were the first reports of photosensitivity.

### Treatment and follow-up

All patients were on antiseizure medications (ASMs). A total of 48.6% (17/35) of patients had three or more ASMs. Two patients (P12, P29) were also treated with ketogenic diets and two patients (P13 and P33) undergone vagus nerve stimulation (VNS), neither of whom had uncontrolled seizures. P27 completed stereo EEG-guided multielectrode stereotactic crossover radiofrequency thermocoagulation with postoperative seizure control. Of note, 77.1% (27/35) of patients received valproate and 55.5% (15/27) of patients received levetiracetam.

At the follow-up after 1 year, six patients (6/10) in PME were followed up and all still had photosensitivity. In *SCN1A*, six patients (6/7) were followed up and three had photosensitivity. In *CHD2*, four patients (4/6) were followed up and only one patient still had photosensitivity. In the other genes, seven patients (7/12) were followed up and all had photosensitivity.

## Discussion

This paper presented the first genetic and clinical analysis of a large cohort of epileptic patients (*n* = 35) with genetic photosensitive to summarize their common features. In our study, ion channel genes (voltage-gated and ligand-gated) accounted for 46.7% (7/15) of single genes, indicating that the dysfunction of ion channels plays a key role in the pathogenesis of photosensitivity.

Previous literature shows that in the general population of photosensitive patients, the most common epileptic syndrome is GGE (3). In our patients with genetically photosensitive, the most common syndromes are PME and Dravet syndrome. There are several reasons that may explain the bias in the data. First seizures in GGE are easily controlled and are not associated with other neurodevelopmental comorbidities, leading to few GGE patients undergoing genetic testing. Second, the underlying genetic architecture of GGE is complex, with different modes of inheritance, including single gene, polygenic, and CNVs (11). Third, the study is a single-center study, and all cases must have an EEG completed at our institution with a PPR or PCR result and a positive genetic test to be enrolled in the study to ensure the reliability of the study.

In our cohort, the top three seizure types were myoclonic seizures, focal seizures, and atypical absence seizures in both spontaneous seizures and PCR. Currently, no relevant similar cohort has been reported. In our study, brain MRI abnormalities were mainly seen in *TPP1, GBA, MFSD8*, and mt-DNA mutations, which were unrelated to photosensitivity but related to the disease itself (e.g., progressive parenchymal atrophy caused by NCLs, abnormal signals in the cerebral cortex caused by mitochondrial disease) (4, 12).

Several scholars have found that low frequencies induced PPRs are often present in CLN2, CLN6 and mitochondrial disease, and have pointed to photosensitivity as an early marker of CLN2 (4, 13). Inspired by these studies, we observed that PPRs at low IPS frequencies were observed in 70% of the PME and PPRs at middle IPS frequencies in 30% of PME patients. Therefore, we can speculate that low-frequency induced PPRs may be a marker of PME. Furthermore, 54.2% (19/35) of our cohort showed low frequencies PPRs and 42.9% (15/35) showed middle frequencies PPRs. Based on the finding, low or middle frequencies PPRs could be considered as one of the most significant features of genetic photosensitivity.

Currently, there are fewer summaries of the common features of EEG in PME. In our PME, 80% of patients had an abnormal background, consistent with previous reports of CNL2 and Unverricht–Lundborg disease (4, 14). Neither the interictal EDs nor the PPR grade were homogeneous, which might be due to the inclusion of a variety of rare heterogeneous disorders in this group. Excluding PPR, other EEG findings in the other patients were not homogeneous.

Photosensitivity is very common in young PME patients (up to 90%), and many genes have been reported to be associated with photosensitivity, including *TPP1, KCTD7, BSCL2, KCNC1, GBA, SCARB2, ASAH1, GOSR2, ATN1, CLN6*, and mt-DNA (3–5, 15–17). In our PME patients, we identified one unreported variant (*MFSD8*) and five previously described variants. The above findings demonstrate that the genes causing PME may be candidates for photosensitivity. According to the HGMD database, more than 40 pathogenic variants in the *MFSD8* genes have been reported, mainly associated with CLN7. In our cohort, the variant of *MFSD8* had been previously reported in the literature, but no photosensitivity was mentioned (18). Moreover, the patient with an *MFSD8* variant had an unusual EEG feature of the posterior EDs for both PPR and interictal EDs.

To date, more than 1,800 *SCN1A* variants have been reported, of which <300 patients with Dravet syndrome have been mentioned for photosensitivity (19, 20). In our cohort, *SCN1A*-related photosensitivity was Dravet syndrome, consistent with previous reports in the literature (20, 21). In 2017, a study showed that the onset and prevalence of PPR was most prominent in the 2nd year of life (20). Among our *SCN1A*-related photosensitive patients, 71.4% (5/7) developed their first PPRs at <3 years of age and earlier than other genes, which also seems to demonstrate the young age of *SCN1A*-related photosentivisity.

To our knowledge, 102 patients carrying 76 different *CHD2* pathogenic or likely pathogenic variants have been reported, including 33 photosensitivity patients (6, 22). In our group, two variants were novel, and the remaining four patients had been previously reported (22). The most common photosensitive syndromes of *CHD2* reported in the literature were Jeavons syndrome, GGE, and Lennox-Gastaut syndrome, all of which were validated in our cohort except for GGE (6, 22). Notably, not only IPS could induce myoclonic seizures in P13 but also startle stimuli (sound) could induce myoclonic seizures, which has never been found in previous patients.

Fifteen variants of *SYNGAP1* have been reported to be photosensitive (23, 24). And our three variants are novel variants, not available in previous literature (23, 24). The characteristic EEGs of *SYNGAP1* variants were: generalized polyspike waves, focal/multifocal discharges, posterior dominant slow waves or bioccipital spikes during ictal or interictal periods, and similar EDs were observed in the EEGs of our three patients (Figure 2d) (25). Currently, patients with the GABA_A_ receptor-related *GABRA1* and *GABRG2* genes, ranging from genetic generalized epilepsy to epileptic encephalopathy, have been found to be photosensitive (26, 27). In our study, the GABA_A_ receptor-related *GABRA1, GABRG2*, and *GRBRB2* genes were found to be possible photosensitive genes. The above findings suggest that the pathophysiology of photosensitivity may be related to GABA inhibitors and that other epilepsy genes of the GABA_A_ receptors may also be photosensitive. Previous studies reported only two cases in one family carrying the same *CACNA1A* variant, and both of them showed PPR on EEG (28). In our patients, P23 carrying a *CACNA1A* variant was mentioned for its photosensitivity in a previous study (29).

In other genes, we identified *KCNMA1, SLC2A1, FLNA*, and *SZT2* as candidate genes. P23 carrying a gain-of-function variant (N955S) of *KCNMA1* was diagnosed with GGE and had a repeat EEG showing PPR or PCR for 3 consecutive years. It has been reported that gain-of function variants of *KCNMA1* could cause GGE, the most common epilepsy in photosensitivity, so it was reasonable to believe that *KCNMA1* might be responsible for photosensitivity (30). Madann et al. reported a pathogenic variant of *SLC2A1* in a family with Jeavons syndrome, where the son's EEG was described as having eye closure sensitivity and photosensitivity (31). Therefore, *SLC2A1*, as the causative gene in the photosensitive model of Jeavons syndrome, might be a candidate gene for photosensitive epilepsy. While *SZT2* and *FLNA* variants were common in epilepsy, there were no reports regarding photosensitivity. This might also be since the standard IPS was overlooked in the EEG examination. The sporadic cases developed photosensitivity, suggesting that further studies are required to investigate the phenotypic variability of the mutations.

Several studies have shown that valproate and levetiracetam appear to be effective in the treatment of photosensitivity (3, 32). Here, valproate was used in 77.1% of patients, and levetiracetam was used in 55.5%. In our cohort, a high proportion (77.7%, 21/27) of photosensitivity was still observed after 1 year of treatment, which suggests that photosensitivity in epilepsy with genetic photosensitivity may have difficulty disappearing in a short period of time.

In summary, we provided a detailed genetic analysis and clinical description of the largest cohort of epilepsy with genetic photosensitivity available. The most common genes for epilepsy with genetic photosensitivity are *SCN1A* and *CHD2*, and the most common syndromes are PME and Dravet syndrome. We identified *MFSD8, KCNMA1*, SZT2, *FLNA*, and *SLC2A1* as candidate genes and indicated that most genes causing PME may be candidates for photosensitivity. A correlation between epilepsy with genetic photosensitivity and IPS was observed: low or middle induced PPRs may the most typical feature of genetic photosensitivity, especially PPRs at low frequencies might be a marker of PME. However, there were still some limitations: the limited number of cases and some unidentified variants. In the future, multicenter studies with genetically related photosensitivity and non-photosensitivity case-control studies could be conducted to reveal the genetic characteristics of epilepsy with photosensitivity.

## Data availability statement

The datasets presented in this study can be found in online repositories. The names of the repository/repositories and accession number(s) can be found in the article/Supplementary material.

## Ethics statement

The studies involving human participants were reviewed and approved by Biomedical Research Ethical Committee of Peking University First Hospital. Written informed consent to participate in this study was provided by the participants' legal guardian/next of kin. Written informed consent was obtained from the individual(s), and minor(s)' legal guardian/next of kin, for the publication of any potentially identifiable images or data included in this article.

## Author contributions

ZY conceptualized and designed the study, coordinated the study overall, and revised the manuscript. YN co-designed the study, drafted the initial manuscript, and revised the manuscript. PG, XJ, ZX, and YZ helped to collect and summarize data and revised the manuscript. All authors approve of the final revision of the article.

## Funding

This work was supported by National Nature Science Foundation of China (81771393 and 82171436), Beijing Natural Science Foundation (7202210), and Capital's Funds for Health Improvement and Research (2020-2-4077).

## Conflict of interest

The authors declare that the research was conducted in the absence of any commercial or financial relationships that could be construed as a potential conflict of interest.

## Publisher's note

All claims expressed in this article are solely those of the authors and do not necessarily represent those of their affiliated organizations, or those of the publisher, the editors and the reviewers. Any product that may be evaluated in this article, or claim that may be made by its manufacturer, is not guaranteed or endorsed by the publisher.

## Footnotes

^1^ http://www.mutationtaster.org/

^2^ http://genetics.bwh.harvard.edu/pph2/

^3^ http://sift.jcvi.org/

^4^ http://www.fruitfly.org/seq_tools/splice.html

^5^ https://cadd.gs.washington.edu/snv
