# Supplementary material for: Genetic and phenotypic spectrum of Chinese patients with epilepsy and photosensitivity
Source: Front Neurol. 2022 Aug 2;13:907228. doi: 10.3389/fneur.2022.907228 (PMC9416002; doi:10.3389/fneur.2022.907228)
Supplement: Supplementary file 1 [file Table_1.docx]

Supplemental materials

**Table S1: The list of genes reported to cause different genetic forms of epilepsy.**

| ***Gene*** | ***Gene*** | ***Gene*** | ***Gene*** | ***Gene*** | ***Gene*** |
| --- | --- | --- | --- | --- | --- |
| ***AARS*** | ***ARHGEF15*** | ***CASR*** | ***COX15*** | ***DPAGT1*** | ***FOLR1*** |
| ***ABAT*** | ***ARHGEF9*** | ***CC2D2A*** | ***COX6B1*** | ***DPM1*** | ***FOXG1*** |
| ***ABCC2*** | ***ARL13B*** | ***CDH13*** | ***CPA6*** | ***DPM3*** | ***FOXP2*** |
| ***ABCC8*** | ***ARSA*** | ***CDH9*** | ***CPLX1*** | ***DPYD*** | ***FOXRED1*** |
| ***ACADSB*** | ***ARSB*** | ***CDKL5*** | ***CPS1*** | ***DRD2*** | ***FRRS1L*** |
| ***ACOX1*** | ***ARSE*** | ***CEP152*** | ***CPT1A*** | ***DRD3*** | ***FUCA1*** |
| ***ACTB*** | ***ARV1*** | ***CEP290*** | ***CPT2*** | ***DTNBP1*** | ***G6PD*** |
| ***ACY1*** | ***ARX*** | ***CHD2*** | ***CSTB*** | ***EBP*** | ***GABBR2*** |
| ***ADAM22*** | ***ASAH1*** | ***CHD7*** | ***CTNNA3*** | ***ECM1*** | ***GABRA1*** |
| ***ADCY5*** | ***ASPA*** | ***CHI3L1*** | ***CTSA*** | ***EEF1A2*** | ***GABRA6*** |
| ***ADGRG1*** | ***ATIC*** | ***CHRNA2*** | ***CTSD*** | ***EFHC1*** | ***GABRB1*** |
| ***ADGRV1*** | ***ATN1*** | ***CHRNA3*** | ***CTSF*** | ***EHMT1*** | ***GABRB2*** |
| ***ADK*** | ***ATP13A2*** | ***CHRNA4*** | ***CUL4B*** | ***EIF2B1*** | ***GABRB3*** |
| ***ADSL*** | ***ATP13A4*** | ***CHRNA5*** | ***CYB5R3*** | ***EIF2B2*** | ***GABRD*** |
| ***AFG3L2*** | ***ATP1A2*** | ***CHRNA7*** | ***CYBB*** | ***EIF2B3*** | ***GABRG2*** |
| ***AGA*** | ***ATP1A3*** | ***CHRNB2*** | ***CYFIP2*** | ***EIF2B4*** | ***GALC*** |
| ***AHI1*** | ***ATP2A2*** | ***CLCN2*** | ***CYP2R1*** | ***EIF2B5*** | ***GALNS*** |
| ***AKT1*** | ***ATP5F1A*** | ***CLCN4*** | ***CYP2U1*** | ***ELP4*** | ***GAMT*** |
| ***ALDH4A1*** | ***ATP6AP2*** | ***CLCNKA*** | ***D2HGDH*** | ***EMX2*** | ***GATAD2B*** |
| ***ALDH5A1*** | ***ATP7A*** | ***CLCNKB*** | ***DAO*** | ***EPB41L1*** | ***GATM*** |
| ***ALDH7A1*** | ***ATPAF2*** | ***CLN3*** | ***DAOA*** | ***EPHB2*** | ***GBA*** |
| ***ALG1*** | ***ATRX*** | ***CLN5*** | ***DBH*** | ***EPM2A*** | ***GCDH*** |
| ***ALG11*** | ***ATXN10*** | ***CLN6*** | ***DBT*** | ***ERBB4*** | ***GCK*** |
| ***ALG12*** | ***B4GALT1*** | ***CLN8*** | ***DCX*** | ***ERLIN2*** | ***GCSH*** |
| ***ALG13*** | ***BANK1*** | ***CNPY3*** | ***DDC*** | ***ETFA*** | ***GFAP*** |
| ***ALG2*** | ***BCKDHA*** | ***CNTN5*** | ***DDOST*** | ***ETFB*** | ***GLB1*** |
| ***ALG3*** | ***BCKDHB*** | ***CNTNAP2*** | ***DENND5A*** | ***ETFDH*** | ***GLDC*** |
| ***ALG6*** | ***BCKDK*** | ***COA5*** | ***DEPDC5*** | ***EVC*** | ***GLRA1*** |
| ***ALG8*** | ***BCS1L*** | ***COG1*** | ***DGKD*** | ***FADD*** | ***GLRB*** |
| ***ALG9*** | ***BOLA3*** | ***COG4*** | ***DHCR7*** | ***FASN*** | ***GLUD1*** |
| ***ALPL*** | ***BRAF*** | ***COG5*** | ***DHFR*** | ***FASTKD2*** | ***GLUL*** |
| ***AMACR*** | ***BSN*** | ***COG6*** | ***DIAPH3*** | ***FBP1*** | ***GNAO1*** |
| ***AMER1*** | ***BTD*** | ***COG7*** | ***DISC1*** | ***FCGR2B*** | ***GNE*** |
| ***AMT*** | ***C12orf65*** | ***COG8*** | ***DLD*** | ***FGD1*** | ***GNPTAB*** |
| ***AP3B2*** | ***C4A*** | ***COL18A1*** | ***DMPK*** | ***FGF12*** | ***GNPTG*** |
| ***AP4S1*** | ***CACNA1A*** | ***COL1A1*** | ***DNAJC5*** | ***FGF8*** | ***GNS*** |
| ***APOL2*** | ***CACNA1H*** | ***COL4A1*** | ***DNAJC6*** | ***FGFR3*** | ***GOSR2*** |
| ***APOL4*** | ***CACNB4*** | ***COMT*** | ***DNASE1*** | ***FH*** | ***GPC3*** |
| ***APP*** | ***CACNG2*** | ***COQ2*** | ***DNM1*** | ***FKRP*** | ***GPHN*** |
| ***APTX*** | ***CAD*** | ***COQ8A*** | ***DOCK6*** | ***FKTN*** | ***GRIA3*** |
| ***ARG1*** | ***CARS2*** | ***COQ9*** | ***DOCK7*** | ***FLNA*** | ***GRIN1*** |
| ***ARHGAP31*** | ***CASK*** | ***COX14*** | ***DOLK*** | ***FLVCR2*** | ***GRIN2A*** |

| ***Gene*** | ***Gene*** | ***Gene*** | ***Gene*** | ***Gene*** | ***Gene*** |
| --- | --- | --- | --- | --- | --- |
| ***GRIN2B*** | ***KCNC2*** | ***MDH2*** | ***NDUFV2*** | ***PEX14*** | ***PRRT2*** |
| ***GRIN2D*** | ***KCNH1*** | ***ME2*** | ***NECAP1*** | ***PEX16*** | ***PSAP*** |
| ***GRN*** | ***KCNH5*** | ***MECP2*** | ***NEDD4L*** | ***PEX19*** | ***PSAT1*** |
| ***GSS*** | ***KCNJ1*** | ***MED12*** | ***NEU1*** | ***PEX2*** | ***PTCH1*** |
| ***GUF1*** | ***KCNJ10*** | ***MEF2C*** | ***NF1*** | ***PEX26*** | ***PTPN11*** |
| ***GUSB*** | ***KCNJ11*** | ***MFSD8*** | ***NF2*** | ***PEX3*** | ***PTPN22*** |
| ***GYS1*** | ***KCNMA1*** | ***MGAT2*** | ***NGLY1*** | ***PEX5*** | ***PUS1*** |
| ***HAX1*** | ***KCNQ1*** | ***MLC1*** | ***NHLRC1*** | ***PEX6*** | ***QDPR*** |
| ***HCN1*** | ***KCNQ2*** | ***MMACHC*** | ***NHS*** | ***PEX7*** | ***RAB39B*** |
| ***HDAC4*** | ***KCNQ3*** | ***MMUT*** | ***NID2*** | ***PGAP2*** | ***RAB3GAP1*** |
| ***HEXA*** | ***KCNT1*** | ***MOCS1*** | ***NOTCH3*** | ***PGAP3*** | ***RAF1*** |
| ***HEXB*** | ***KCNT2*** | ***MOCS2*** | ***NPC1*** | ***PGK1*** | ***RANBP2*** |
| ***HFE*** | ***KCTD7*** | ***MOCS3*** | ***NPC2*** | ***PGM1*** | ***RARS2*** |
| ***HGSNAT*** | ***KDM5C*** | ***MOGS*** | ***NPHP1*** | ***PHF6*** | ***RBFOX1*** |
| ***HLA-DQA1*** | ***KIF11*** | ***MPC1*** | ***NPHS1*** | ***PHGDH*** | ***RBFOX2*** |
| ***HLA-DQB1*** | ***KIF1A*** | ***MPDU1*** | ***NR3C1*** | ***PIGA*** | ***RBFOX3*** |
| ***HNF1B*** | ***KMT2D*** | ***MPI*** | ***NRAS*** | ***PIGL*** | ***RELN*** |
| ***HNRNPH1*** | ***KRAS*** | ***MR1*** | ***NRXN1*** | ***PIGN*** | ***RFT1*** |
| ***HNRNPU*** | ***KRIT1*** | ***MTHFR*** | ***NTNG1*** | ***PIGO*** | ***RHOBTB2*** |
| ***HP*** | ***L2HGDH*** | ***MTOR*** | ***NTRK2*** | ***PIGP*** | ***RNASEH2A*** |
| ***HPD*** | ***LAMA2*** | ***MTR*** | ***NUBPL*** | ***PIGV*** | ***RNASEH2B*** |
| ***HRAS*** | ***LARGE1*** | ***MTRR*** | ***OFD1*** | ***PIGW*** | ***RNASEH2C*** |
| ***HSD17B10*** | ***LBR*** | ***NAGLU*** | ***OPA1*** | ***PIGY*** | ***ROGDI*** |
| ***HSD17B4*** | ***LGI1*** | ***NDE1*** | ***OPHN1*** | ***PLA2G6*** | ***RPGRIP1L*** |
| ***HTR2A*** | ***LGR4*** | ***NDN*** | ***OTC*** | ***PLCB1*** | ***RPIA*** |
| ***HTT*** | ***LIAS*** | ***NDUFA1*** | ***PAFAH1B1*** | ***PLP1*** | ***RTN4R*** |
| ***HYAL1*** | ***LIG4*** | ***NDUFA11*** | ***PAH*** | ***PLPBP*** | ***RYR1*** |
| ***IDH2*** | ***LMX1B*** | ***NDUFA2*** | ***PAK3*** | ***PMM2*** | ***RYR3*** |
| ***IDS*** | ***LRPPRC*** | ***NDUFAF1*** | ***PANK2*** | ***PNKD*** | ***SAMHD1*** |
| ***IDUA*** | ***MAGI1*** | ***NDUFAF2*** | ***PAX6*** | ***PNKP*** | ***SCARB2*** |
| ***IER3IP1*** | ***MAGI2*** | ***NDUFAF3*** | ***PC*** | ***PNPO*** | ***SCN1A*** |
| ***IFNG*** | ***MAGT1*** | ***NDUFAF4*** | ***PCCA*** | ***POLG*** | ***SCN1B*** |
| ***IL6*** | ***MAN1B1*** | ***NDUFAF5*** | ***PCDH19*** | ***POMGNT1*** | ***SCN2A*** |
| ***INPP5E*** | ***MANBA*** | ***NDUFB3*** | ***PCNT*** | ***POMT1*** | ***SCN3A*** |
| ***INS*** | ***MAP2K1*** | ***NDUFS1*** | ***PDHA1*** | ***POMT2*** | ***SCN4A*** |
| ***INSR*** | ***MAP2K2*** | ***NDUFS2*** | ***PDHX*** | ***PPOX*** | ***SCN8A*** |
| ***IQSEC2*** | ***MAPK10*** | ***NDUFS3*** | ***PDSS1*** | ***PPT1*** | ***SCN9A*** |
| ***ITPA*** | ***MBD5*** | ***NDUFS4*** | ***PDSS2*** | ***PQBP1*** | ***SCO2*** |
| ***KCNA1*** | ***MCCC2*** | ***NDUFS6*** | ***PEX1*** | ***PRICKLE1*** | ***SDHA*** |
| ***KCNA2*** | ***MCOLN1*** | ***NDUFS7*** | ***PEX10*** | ***PRICKLE2*** | ***SERPINI1*** |
| ***KCNB1*** | ***MCPH1*** | ***NDUFS8*** | ***PEX12*** | ***PROC*** | ***SETBP1*** |
| ***KCNC1*** | ***MDGA2*** | ***NDUFV1*** | ***PEX13*** | ***PRODH*** | ***SGCE*** |
| ***SGSH*** | ***SLC25A22*** | ***SOS1*** | ***SYN1*** | ***TPP1*** | ***UNC80*** |
| ***SHANK3*** | ***SLC26A4*** | ***SPAST*** | ***SYN2*** | ***TREM2*** | ***VPS13A*** |
| ***SHH*** | ***SLC2A1*** | ***SPTAN1*** | ***SYNGAP1*** | ***TREX1*** | ***VPS13B*** |
| ***SHOC2*** | ***SLC35A1*** | ***SPTLC2*** | ***SYNJ1*** | ***TRPM6*** | ***VRK1*** |
| ***Gene*** | ***Gene*** | ***Gene*** | ***Gene*** | ***Gene*** | ***Gene*** |
| ***SIK1*** | ***SLC35A2*** | ***SRD5A3*** | ***SYP*** | ***TSC1*** | ***VRK2*** |
| ***SIX3*** | ***SLC35C1*** | ***SRPX2*** | ***SZT2*** | ***TSC2*** | ***VWF*** |
| ***SLC12A5*** | ***SLC46A1*** | ***ST3GAL2*** | ***TACO1*** | ***TSEN2*** | ***WDR45*** |
| ***SLC13A5*** | ***SLC6A1*** | ***ST3GAL3*** | ***TBC1D24*** | ***TSEN34*** | ***WWOX*** |
| ***SLC16A2*** | ***SLC6A8*** | ***ST3GAL5*** | ***TBP*** | ***TSEN54*** | ***XK*** |
| ***SLC17A5*** | ***SLC9A6*** | ***STRADA*** | ***TBX1*** | ***TUBA1A*** | ***YWHAG*** |
| ***SLC19A3*** | ***SLC9A9*** | ***STS*** | ***TCF4*** | ***TUBG1*** | ***ZDHHC15*** |
| ***SLC1A2*** | ***SMC1A*** | ***STX1B*** | ***TH*** | ***TUBGCP6*** | ***ZEB2*** |
| ***SLC1A3*** | ***SMPD1*** | ***STXBP1*** | ***TMEM165*** | ***TUSC3*** | ***ZFYVE26*** |
| ***SLC20A2*** | ***SMS*** | ***SUCLA2*** | ***TMEM216*** | ***TWNK*** | ***ZNF41*** |
| ***SLC25A12*** | ***SNIP1*** | ***SUMF1*** | ***TMEM67*** | ***TYROBP*** |  |
| ***SLC25A15*** | ***SNRPN*** | ***SUOX*** | ***TMEM70*** | ***UBA5*** |  |
| ***SLC25A19*** | ***SOBP*** | ***SURF1*** | ***TNK2*** | ***UBE3A*** |  |
